# Breaking Latent Infection: How ORF37/38-Deletion Mutants Offer New Hope against EHV-1 Neuropathogenicity

**DOI:** 10.3390/v16091472

**Published:** 2024-09-16

**Authors:** Yue Hu, Si-Yu Zhang, Wen-Cheng Sun, Ya-Ru Feng, Hua-Rui Gong, Duo-Liang Ran, Bao-Zhong Zhang, Jian-Hua Liu

**Affiliations:** 1 Laboratory of Animal Infectious Disease, College of Veterinary Medicine, Xinjiang Agricultural University, Urumqi 830052, China; y.hu1@siat.ac.cn (Y.H.); ws.yhxnd@outlook.com (W.-C.S.); rs.xny@outlook.com (Y.-R.F.); dr.xjxnd@outlook.com (D.-L.R.); 2 CAS Key Laboratory of Quantitative Engineering Biology, Shenzhen Institutes of Advanced Technology (SIAT), Chinese Academy of Sciences, Shenzhen 518055, China

**Keywords:** equid alphaherpesvirus 1, latent infection, neuropathogenicity, attenuated vaccines

## Abstract

Equid alphaherpesvirus 1 (EHV-1) has been linked to the emergence of neurological disorders, with the horse racing industry experiencing significant impacts from outbreaks of equine herpesvirus myeloencephalopathy (EHM). Building robust immune memory before pathogen exposure enables rapid recognition and elimination, preventing infection. This is crucial for effectively managing EHV-1. Removing neuropathogenic factors and immune evasion genes to develop live attenuated vaccines appears to be a successful strategy for EHV-1 vaccines. We created mutant viruses without ORF38 and ORF37/38 and validated their neuropathogenicity and immunogenicity in hamsters. The ∆ORF38 strain caused brain tissue damage at high doses, whereas the ∆ORF37/38 strain did not. Dexamethasone was used to confirm latent herpesvirus infection and reactivation. Dexamethasone injection increased viral DNA load in the brains of hamsters infected with the parental and ∆ORF38 strains, but not in those infected with the ∆ORF37/38 strain. Immunizing hamsters intranasally with the ∆ORF37/38 strain as a live vaccine produced a stronger immune response compared to the ∆ORF38 strain at the same dose. The hamsters demonstrated effective protection against a lethal challenge with the parental strain. This suggests that the deletion of ORF37/38 may effectively inhibit latent viral infection, reduce the neuropathogenicity of EHV-1, and induce a protective immune response.

## 1. Introduction

Equid alphaherpesvirus 1 (EHV-1) is a common virus that has been demonstrated to induce a range of clinical signs in equids, including respiratory disease, abortion, and neurological signs [1]. Recently, equine herpesvirus myeloencephalopathy (EHM) has increased in various regions [2,3]. The neurological manifestations observed in affected horses range from mild ataxia to paraplegia [4]. The prognosis for horses with EHM who experience recurrent episodes or require support in a sling is often considered unfavorable. These viral outbreaks have resulted in significant economic losses to the racing industry [5,6].

During outbreaks of equine respiratory disease by EHV-1 infection, EHM (equine herpes myeloencephalopathy) may manifest during the initial infection or reappear upon reactivation of the virus from latency and subsequent reinfection [7]. In some instances, EHM does not become apparent during the initial infection; however, EHV-1 can evade the innate immune system, establishing a latent infection in nerve ganglia [8]. The virus can then periodically reactivate from latency, leading to the onset of EHM. Establishing a robust immune memory prior to the primary infection enables the host to rapidly recognize and clear the pathogen, thereby preventing both acute and latent infections, which is critical for the control of EHM [9]. Developing a vaccine for herpesviruses is difficult due to immune evasion genes and the latent phase [10]. The development of live attenuated vaccines through the removal of neuropathogenic factor and viral immune evasion genes, to enhance immunogenicity and guarantee safety, seems to be a successful approach for the formulation of EHV-1 vaccines [11]. 

Previous studies have indicated that single-nucleotide polymorphisms (SNPs) within the EHV1 ORF30 gene of the DNA polymerase of EHV-1 are strongly associated with neurological signs [12]. The ORF30 D752 variant is referred to as the neuropathogenic type, while the ORF30 N752 variant is referred to as the non-neuropathogenic type [13]. In 2020, a novel variant (ORF30 H752) was identified in France, which was not thought to be associated with neurological signs [14]. Nevertheless, in our previous study, an ORF30 N752 variant that had previously been considered non-neuropathogenic was isolated and shown to induce characteristic neurological signs in infected horses and Syrian hamsters [15]. This finding suggested that there might be other factors that enhance the neuropathogenicity of the virus. In studies related to EHV-1 neuropathogenicity, ORF37 has been identified as a potential contributor to mouse encephalitis, although the exact mechanism involved remains unclear [16]. In contrast, in other herpesviruses, ORF37 is considered to be linked to the virus’s latent state and subsequent reactivation [17]. Furthermore, ORF38 in the herpesvirus family has been demonstrated to play a pivotal role in latent viral infection and virulence [18]. This gene encodes an enzymatically active nonstructural protein (ORF38) that phosphorylates deoxyribonucleosides and promotes viral DNA replication and transcription [19]. It is widely accepted that alphaherpesvirus with deletions or mutations in ORF38 is unable to establish latent infection in ganglia and exhibits reduced virulence [20,21].

In this study, we constructed a ∆ORF37/38 double-deletion strain from a neuropathogenic EHV-1 strain (ORF30 N752). This deletion strain was unable to cause neuropathogenicity in Syrian hamsters. Despite stimulation with dexamethasone, no evidence of the reactivation of latent infection was observed in the brains of the hamsters. Furthermore, the immunization of hamsters with the ∆ORF37/38 virus elicited both humoral and cellular immune responses against EHV-1, resulting in the complete protection of hamsters from exposure to lethal challenge with the parental strain. These findings suggested that the deletion of ORF37/38 could effectively attenuate the neuropathogenicity and reactivation of EHV-1 while also eliciting a protective immune response.

## 2. Materials and Methods

### 2.1. Virus, Cell Lines, Primers, and Plasmids

The EHV-1 YM2019 strain was preserved at the China General Microbiological Culture Collection Center, and its genomic sequence is deposited in GenBank under the accession number MT063054 [15]. Rabbit kidney epithelial cells (RK-13, ATCC CCL-37) were obtained from the American Type Culture Collection. Homologous donor plasmids (HDR-ORF38 and HDR-ORF37/38) and homologous donor plasmids containing eGFP with the CMV promoter (HDR-ORF38-eGFP and HDR-ORF37/38-eGFP) were constructed using the Ezmax one-step cloning kit (Tolo Biotech). All the primers for PCR and sgRNA are shown in Supplementary Table S1. Supplementary Table S2 contains a list of the strains and plasmids used in this study.

### 2.2. Construction of EHV-1 ∆ORF38 and ∆ORF37/38 Mutants

The YM2019 ∆ORF38 and ∆ORF37/38 strains were constructed using CRISPR/Cas9 gene editing and homologous recombination methods, as described previously [22], with some modifications and appropriate primers (Supplementary Table S1).

As shown in Supplementary Figure S1, first, a single guide RNA (sgRNA) targeting ORF38 was used to guide the CRISPR/Cas9 protein to cleave the ORF38 gene. The HDR-ORF38-eGFP construct served as a template for homology-directed repair (HDR), facilitating spontaneous HDR. Plaque purification was performed for five rounds, and recombinant viruses emitting green fluorescence under a fluorescence microscope were selected. The viral genome sequences were validated by PCR and sequencing techniques, confirming that the ORF38 gene sequences had been replaced with an eGFP gene expression cassette. Subsequently, an sgRNA targeting eGFP guided the CRISPR/Cas9 protein to cleave the eGFP gene. HDR-ORF38 served as the template for HDR, and plaque purification was performed for five rounds, selecting recombinant viruses that did not emit green fluorescence under a fluorescence microscope. The viral genome sequences were again validated by PCR and sequencing techniques, confirming that both the ORF38 and eGFP genes had been deleted.

As shown in Supplementary Figure S2, two sgRNAs targeting ORF37 and ORF38 simultaneously guided the CRISPR/Cas9 protein to cleave both genes. The HDR-ORF37/38-eGFP construct served as a template for HDR, replacing the ORF37 and ORF38 genes with an eGFP expression cassette. The recombinant virus that emits green fluorescence was screened and purified. Then, an sgRNA targeting eGFP guided the CRISPR/Cas9 protein to cleave the eGFP gene. HDR-ORF37/ORF38 served as the template for HDR, and non-fluorescent recombinant viruses were selected and purified. The viral genome sequences were validated by PCR and sequencing techniques, confirming that both the ORF37/ORF38 genes and the eGFP gene had been deleted.

### 2.3. Viral Growth Kinetics

Plaque sizes were quantified 48 h after inoculation with the virus at a multiplicity of infection (MOI) of 0.1 [23]. After a 1 h incubation period with the virus, the medium was replaced with a 1% low-melting-point agarose overlay containing 2% fetal bovine serum (FBS) in Dulbecco’s Modified Eagle Medium (DMEM) to promote plaque formation. The dimensions of 100 plaques per viral strain were measured using the NIS-Elements Viewer software v.4.50 (Nikon Group). These measurements were standardized relative to the dimensions of the EHV-1 YM2019 strain, which served as the baseline at 100%. The mean percentages and standard deviations were calculated from four independent experiments. 

The growth kinetics of the deletion mutant viruses were assessed by measuring growth curves at the one-step growth curve. RK-13 cells were infected with the WT, ∆ORF38, and ∆ORF37/38 strains at an MOI of 0.1, as described in reference [24]. Cells infected with these strains were collected at predetermined time (at 6, 12, 24, 36, 48, 60, and 72 h post-infection) intervals to determine viral titers using the 50% tissue culture infectious dose (TCID_50_) assay. The viral titers were calculated using the Reed–Muench method. The replication kinetics curves were constructed based on data from four independent experiments.

### 2.4. Evaluation of Neuropathogenicity

Forty-eight female specific pathogen-free (SPF) Syrian hamsters, aged 4 weeks, were procured from Charles River Laboratories (CRL) and randomly allocated into four groups (Numbers (n) = 12). The infection groups were intranasally administered 0.1 mL of PBS containing 10^8^ TCID_50_ of the viruses. The control group hamsters received an inoculation of 0.1 mL of sterile PBS. 

Hamsters (n = 6) were observed twice daily for 14 days: body weight, survival, and clinical scores were recorded. Clinical scores were assigned according to a 15-point scoring system, shown in Supplementary Table S3 [25]: a score of < 3 indicates no clinical signs; 3 ≤ score < 5 indicates mild clinical signs; 5 ≤ score < 8 indicates moderate clinical signs; 8 ≤ score < 10 indicates severe clinical signs; a score of > 10 indicates fatal signs. The animals were euthanized if necessary to alleviate distress. During euthanasia with isoflurane anesthesia (5%), hamsters were cervical dislocated and euthanized.

At 8 days post-inoculation (dpi), samples (n = 6) were collected from the brain, lungs, and lymph nodes of each hamster in the infection groups for pathological examination and viral load analysis. Histopathological examination was performed utilizing hematoxylin–eosin (HE) staining [26]. Histopathologic scoring was conducted on lung and brain tissues in accordance with established principles [27]. Lung tissue was assessed for five lesion parameters, including interstitial pneumonia, fibrosis, inflammatory exudates, neutrophilic inflammation, and hemorrhage. Brain tissue was evaluated for nonsuppurative encephalitis, neuronal necrosis, macrophage infiltration, microglial activation, and gliosis. Each parameter was assigned a score on an ordinal scale of 0 (normal), 1 (mild), 2 (moderate), and 3 (severe) to indicate the severity of the lesions. The total histopathology scores for lung and brain tissues in each hamster were 15 points (Supplementary Table S3). The viral DNA load was assessed through real-time PCR targeting ORF68 using primers and a specific probe [28,29].

### 2.5. Evaluation of the Latency of Reactivation

Sixty female SPF Syrian hamsters aged 4 weeks were randomly allocated into four groups. The infection groups (n = 18) were intranasally administered 0.1 mL of PBS containing either 10^4^ TCID_50_ (WT strain) or 10^5^ TCID_50_ (∆ORF38 and ∆ORF37/38 strains) of the viruses. The control group (n = 6) hamsters received an inoculation of 0.1 mL of sterile PBS. At 21 days post-infection (dpi), the hamsters were administered 100 mg/kg of dexamethasone (DEX) intramuscularly, and 20 mg/kg of DEX was administered at 22 and 23 dpi [30]. Body weights and clinical signs of morbidity were monitored daily. At 5 and 10 days after DEX stimulation, the hamsters were euthanized and necropsied, and pathological scores and viral DNA loads were obtained.

### 2.6. Evaluation of Immune Efficacy

Sixty female SPF Syrian hamsters were randomly assigned to five groups, consisting of four vaccinated groups and a control group (n = 12). The vaccinated groups were intranasally administered 0.1 mL of PBS containing either 10^8^ TCID_50_, 10^7^ TCID_50_, or 10^6^ TCID_50_ of the ∆ORF37/38 strain or 10^7^ TCID_50_ of the ∆ORF38 strain. The control group hamsters received an inoculation of 0.1 mL of PBS. At 21 days post-vaccination (dpv), hamsters in both the vaccinated and control groups were challenged via intranasal delivery of 10^8.15^ TCID_50_ YM2019. At 14 days post-challenge (dpc), the surviving hamsters were euthanized and necropsied, and various organ samples were collected for pathologic examination and viral load testing.

### 2.7. Serological Test

Serum samples were obtained at 0, 7, 14, 21, 28, and 35 dpv. The levels of anti-gG autoantibodies (IgG) in the serum were quantified using an indirect enzyme-linked immunosorbent assay (I-ELISA) [29]. Viral neutralization assays were performed as described previously [31]. Serum samples were heat-inactivated at 56 °C for 30 min. Fifty microliters of diluted sera was incubated with 50 μL of virus (200 TCID_50_) for 1 h at 37 °C. The mixture was then added to RK-13 cells in a 96-well plate. The cells were cultured for 4 days and assessed for cytopathic effects (CPEs) under a microscope. The titers of neutralizing antibodies were calculated as the reciprocals of the highest serum dilutions at which no CPE was observed. The peak values were determined using area under the curve (AUC) analyses [32].

### 2.8. Cell-Mediated Immune Responses

As described above, thirty female SPF Syrian hamsters were randomly assigned to five groups, consisting of four vaccinated groups and a control group (n = 6). The hamsters were euthanized at 21 dpv to harvest splenocytes. Using an MTT-based assay [33], cells were plated in 96-well plates with 100 μL of media containing the inactivated EHV-1 antigen and cultured for 72 h. The splenocytes were plated in 6-well plates and stimulated with media supplemented with EHV-1 for 24 h after seeding. Cytokine levels were quantified in spleen extracts from hamsters using commercial ELISA kits (Mouse IFNγ and IL-10 ELISA MAX Standard kits (BioLegend)), following the manufacturer’s instructions [34]. 

## 3. Results

### 3.1. The ∆ORF37/38-Gene-Deletion Virus Does Not Induce Neurological Signs in Hamsters during Acute Infection

**Figure 1:**
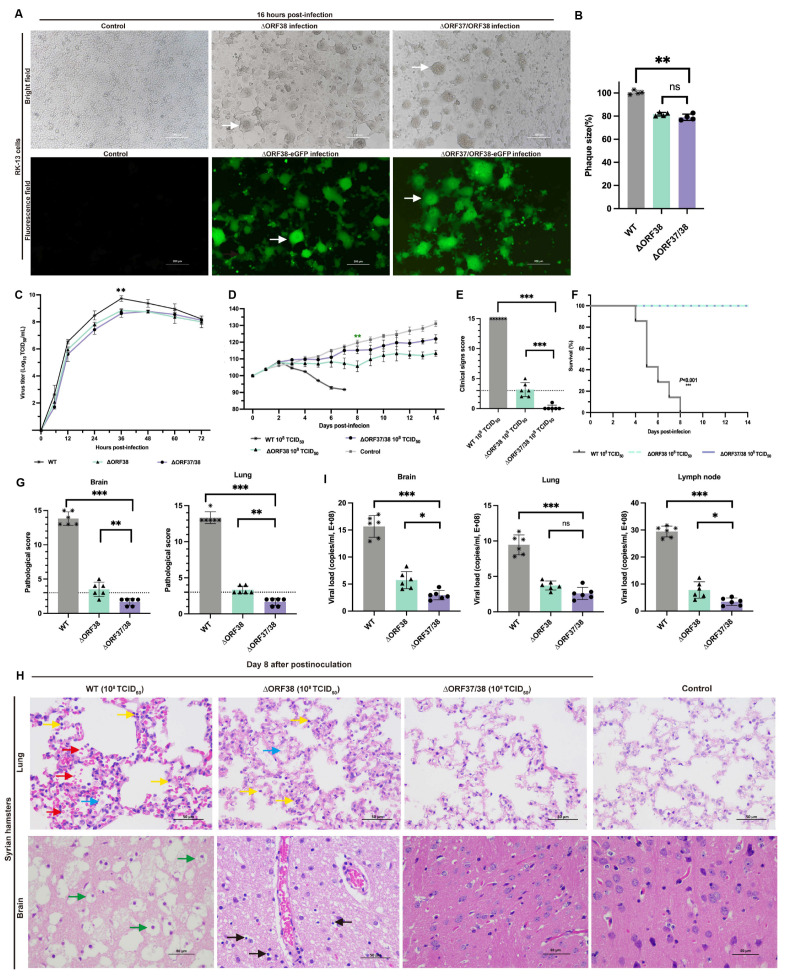
Replication properties in vitro and pathogenicity in hamsters of ∆ORF38 and ∆ORF37/38. (**A**) Herpes-characteristic CPEs (syncytium formation, rounded cells) were observed after infecting RK-13 cells with ∆ORF38, ∆ORF37/38, ∆ORF38-eGFP, and ∆ORF37/38-eGFP strains at an MOI of 0.01 for 16 h, (fluorescence microscope, emission filter bandpass, 505–530 nm); the white arrow points to the syncytium. (**B**) The mean plaque size of each virus is shown as a percentage relative to the WT plaque size. (**C**) One-step growth curve. (**D**) Body-weight loss of hamsters within 14 days post-infection (n = 6). Green asterisks indicate a significant difference (∆ORF38-infected group compared to the control group) ** *p* < 0.01. (**E**) Clinical sign scoring. The dashed line represents a clinical sign score of 3; scores above 3 indicate the presence of typical clinical signs. (**F**) Survival curve. (**G**) Pathological scoring of lung and brain tissue. The dashed line represents a pathological score of 3; scores above 3 indicate the presence of pathological damage. (**H**) Hematoxylin and eosin (HE) staining was utilized to detect pathological lesions. Blue markings indicate alveolar inflammatory exudates, red markings indicate hemorrhage, yellow markings indicate infiltration of inflammatory cells, green markings indicate vacuolated neurons, and black markings indicate activation of microglia. (**I**) Viral load in different tissues of infected hamsters. Statistical differences were analyzed by one-way ANOVA. * *p* < 0.05, ** *p* < 0.01, *** *p* < 0.001, ns *p* > 0.05.

CRISPR-Cas9 gene editing technology was used to knock out ORF37 and ORF38. ORF37/38 were initially replaced with an eGFP gene expression cassette to ensure complete deletion of the ORF37/38 genes. This was followed by the complete deletion of the eGFP expression cassette. The sequence of the purified virus was then verified by Sanger sequencing. RK-13 cells infected separately with ∆ORF38 and ∆ORF37/38 strains exhibited Herpes virus characteristic cytopathic effects (CPEs). Additionally, CPEs and green fluorescence were also observed in cells infected separately with ∆ORF38-eGFP and ∆ORF37/38-eGFP recombination (Figure 1A). Compared with those of the parental virus, the mean plaque sizes of the ∆ORF38 and ∆ORF37/38 viruses were slightly smaller (*p* < 0.01) (Figure 1B). The one-step growth curve indicated that the parental virus exhibited the highest titer of 10^9.73^ TCID_50_/mL at 36 h, while the ∆ORF38 and ∆ORF37/38 viruses achieved titers of 10^8.84^ TCID_50_/mL and 10^8.78^ TCID_50_/mL, respectively, at the same time point (*p* < 0.01) (Figure 1C).

The pathogenicity of the ∆ORF37/38 recombinant virus was verified in a Syrian hamster infection model. Hamsters were infected by intranasal injection of 10^8^ TCID_50_ of the virus for 14 days without significant weight loss or clinical signs, and none of the hamsters died. In contrast, the ∆ORF38 strain-infected animals exhibited significant weight loss (*p* < 0.01) (Figure 1D). Two hamsters exhibited clinical signs characterized by standing still, a small amount of watery nasal discharge, and increased rate, which were clinically scored as mild and moderate clinical signs, respectively (Figure 1E).

No lethality was observed with the mutants; however, all hamsters infected with the parental virus at the same infectious dose succumbed to the infection within eight days (Figure 1F). The hamsters displayed signs of respiratory distress, circling behavior, and sudden changes in body posture, including flexion of the trunk and tonic extension of the limbs, or hyperextension of the back with tonic posturing of the limbs, before succumbing to death (Supplementary Figure S3). At 8 dpi, lung and brain tissues were obtained for pathological analysis. Gross pathological observations of the lungs in hamsters infected with the parental strain included pulmonary congestion and edema, with a mottled appearance on the surface, accompanied by exudation of fibrin and red blood cells (Supplementary Figure S4). Microscopic examination revealed interstitial pneumonia with markedly reduced alveolar spaces, along with interstitial fibroproliferation and hemorrhage. The alveolar walls were thickened, accompanied by infiltration of neutrophils and lymphocytes. Pathological scores were all above 10, indicating severe pathological damage (Figure 1G). Gross pathological lesions in the brains of hamsters infected with the parental strain presented as mild congestion and edema (Supplementary Figure S4). Microscopic examination indicated the presence of nonsuppurative encephalitis, extensive neuronal necrosis, and activation of microglial cells. Pathological scores were all above 10, indicating severe pathological damage (Figure 1G).

Compared with those from the hamsters infected with the parental virus, the lung tissues of the hamsters in the ΔORF38 strain-infected group exhibited mild inflammatory exudate and inflammatory cell infiltration. Mild neuronal necrosis and activation of microglial cells were observed in the brain tissue (Figure 1H). In contrast, hamsters infected with the ΔORF37/38 strain showed no significant pathological damage, with pathology scores of less than 3 in the brain and lungs (Figure 1G). The viral load in the lungs did not demonstrate a statistically significant difference between hamsters infected with the ∆ORF38 strain and those infected with the ∆ORF37/38 strain (*p* > 0.05) (Figure 1I). Conversely, the viral loads in brain tissue and lymph nodes exhibited a notable disparity between the two groups (*p* < 0.05) (Figure 1I). The results indicate that the deletion of the ORF37/38 genes significantly attenuates the pathogenicity of EHV-1 in acutely infected hamsters.

### 3.2. Inability of the ∆ORF37/38 Virus to Cause an Increase in Clinical Signs or Viral DNA Loads with Dexamethasone Stimulation

**Figure 2:**
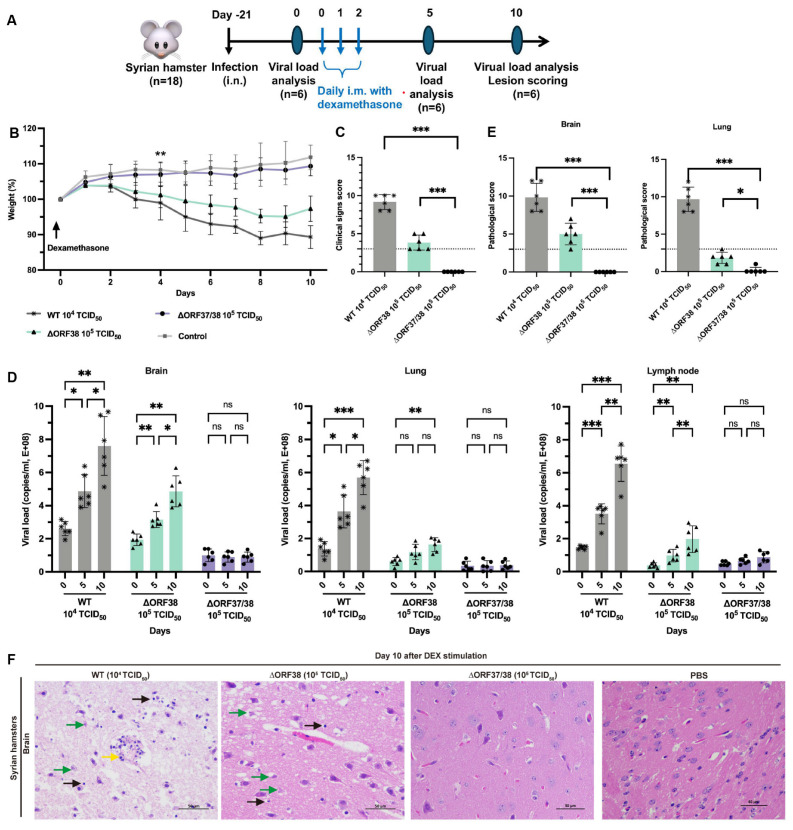
Evaluation of reactivation of ∆ORF38 and ∆ORF37/38. (**A**) Schematic diagram of infection, DEX stimulation, and sample collection in hamsters. (**B**) Body weight. Blank asterisks indicate a significant difference (WT and ∆ORF38 compared to the control group) ** *p* < 0.01. (**C**) Clinical sign scoring. (**D**) Viral DNA load analysis in lung, brain, and lymph nodes at day 0, day 5, and day 10. (**E**) Pathological scoring and (**F**) histopathological lesions in the brain tissues of the hamsters at day 10. Yellow markings indicate perivascular cuffing of macrophage–lymphocyte cells (nonsuppurative encephalitis), green markings indicate necrosis of neurons, and black markings indicate activation of microglia. Statistical differences were analyzed by one-way ANOVA. * *p* < 0.05, ** *p* < 0.01, *** *p* < 0.001, ns *p* > 0.05. The dashed line represents a score of 3.

Modeling the stress response with DEX can effectively reactivate herpesviruses from latency [30]. To ensure effective reactivation, three intramuscular injections of DEX were administered, starting on day 21 following the infection of hamsters with low-dose infectious WT (10^4^ TCID_50_), ∆ORF38 (10^5^ TCID_50_), and ∆ORF37/38 (10^5^ TCID_50_) viruses (Figure 2A). After five days of stimulation, the hamsters in the ∆ORF38 strain- and WT strain-infected groups began to lose weight and exhibited signs of depression (Figure 2B). At ten days post-stimulation, all hamsters in the WT strain 10^4^ TCID_50_-infected group and two hamsters in the ∆ORF38 strain 10^5^ TCID_50_-infected group developed respiratory distress and displayed circling, twitching, and ataxic movements. The clinical scores for these hamsters were all above 3; however, no fatalities were observed (Figure 2C). Tissue samples from the hamsters were obtained for analysis on days five and ten post-stimulation. The viral loads in brain tissues from the WT strain 10^4^ TCID_50_-infected group and the ∆ORF38 strain 10^5^ TCID_50_-infected group increased after five days of stimulation, and then, rapidly increased by day ten (Figure 2D). Furthermore, elevated viral loads were observed in the lung and lymph nodes on day ten. Pathological section analysis of brain tissue in the WT strain 10^4^ TCID_50_-infected group and the ∆ORF38 strain 10^5^ TCID_50_-infected group revealed evidence of pathological damage, whereas the ∆ORF37/38 strain 10^5^ TCID_50_-infected group did not display such indications (*p* < 0.001) (Figure 2E,F). The results indicate that the ∆ORF37/38 mutant did not cause an increase in clinical signs or viral DNA loads in lung and brain tissues following multiple injections of DEX, compared to the WT strain. The ∆ORF37/38-gene-deletion strain showed minimal neuropathogenicity in hamsters.

### 3.3. Intranasal Vaccination with the ∆ORF37/38 Mutant Effectively Protects Hamsters from Lethal Infections

**Figure 3:**
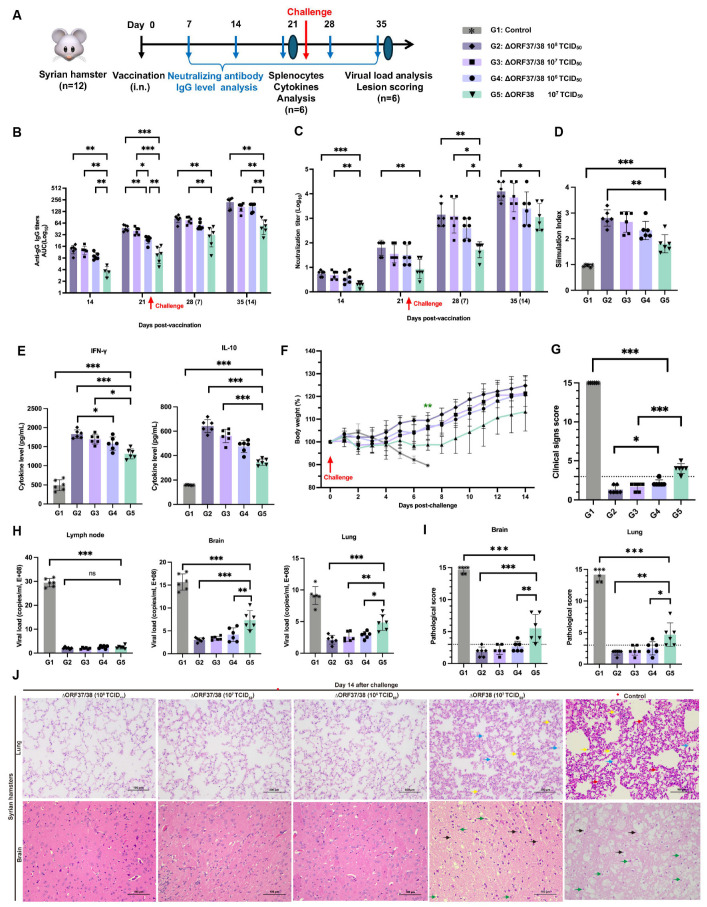
Immunogenicity and protection efficacy of intranasal inoculation with ∆ORF37/38. (**A**) Schematic diagram of vaccination, challenge, and sample collection in hamsters. (**B**) EHV-1 gG specific serum antibody and (**C**) neutralizing antibody titers. Splenocytes from ∆ORF37/38-immunized groups exhibited significantly higher (**D**) stimulation index and (**E**) cytokine levels (IFN-γ and IL-10) when comparing with ∆ORF38-immunized group. (**F**) Body-weight loss was recorded until 14 dpc. Green asterisks indicate a significant difference (∆ORF38-immunized group compared to the ∆ORF37/38-immunized group) ** *p* < 0.01. (**G**) Clinical sign scoring. (**H**) Viral load analysis in lung, brain, and lymph nodes at 14 dpc. (**I**) Lesion scoring and (**J**) histopathological lesions. Blue markings indicate alveolar inflammatory exudates, red markings indicate hemorrhage, yellow markings indicate infiltration of inflammatory cells, green markings indicate necrosis of neurons, and black markings indicate activation of microglia. Statistical differences were determined by one-way ANOVA analysis with Bonferroni’s multiple comparison test. * *p* < 0.05, ** *p* < 0.01, *** *p* < 0.001, ns *p* > 0.05. The dashed line represents a score of 3.

An immunoassay experiment schematic is shown in Figure 3A. The administration of the ∆ORF37/38 virus resulted in the induction of high levels of EHV-1-specific neutralizing antibodies as early as 14 dpv, which was accompanied by the production of IgG antibodies. At all time points, the ∆ORF37/38 strain-immunized groups exhibited greater levels of IgG antibodies and neutralizing antibodies than did the ∆ORF38 strain-immunized group (*p* < 0.05) (Figure 3B,C). Furthermore, the antibody levels exhibited a marked increase following the challenge, with a statistically significant difference between the groups that received the ∆ORF37/38 strain immunization and the ∆ORF38 strain-immunized group at 14 dpc (*p* < 0.01).

The splenic lymphocyte proliferation assay and cytokine levels were used to assess cellular immune responses. Following stimulation with the inactivated EHV-1 antigen, the stimulation indices of all immunized groups were notably greater than the control (*p* < 0.001) and the ∆ORF38 strain-immunized group (*p* < 0.01) (Figure 3D). The results indicate that the vaccination with the ∆ORF37/38 strain promotes the generation of memory lymphocytes capable of proliferating in response to specific antigens. Additionally, hamsters that were immunized with the ∆ORF37/38 strain had more than threefold higher IFN-γ levels than the control (*p* < 0.001), while their levels of IL-10 were elevated approximately twofold (*p* < 0.001) (Figure 3E). Consistent with the antibody levels, immunization with the ∆ORF37/38 strain resulted in elevated cytokine levels compared with immunization with the ∆ORF38 strain, suggesting the successful establishment of T-cell immune responses through immunization. At 21 days after immunization, we administered a lethal dose of the parental virus intranasally. By 14 dpc, no fatalities were recorded among the vaccinated hamsters, whereas all hamsters in the control group succumbed to the challenge within seven days. Hamsters in the ∆ORF38 strain-immunized group experienced weight loss (*p* < 0.01) and depression, but no respiratory distress, neurological signs, or deaths were observed (Figure 3F). The clinical scores for these hamsters were all above 3, which was significantly different from all of the ∆ORF37/38 strain-immunized group (*p* < 0.001) (Figure 3G). The clinical scores of the hamsters in the ∆ORF38 10^8^ TCID_50_-immunized group were all 3 points lower, which was significantly different from the ∆ORF37/38 10^6^ TCID_50_-immunized group (*p* < 0.05) (Figure 3G).

At 14 dpc, there was a highly significant difference in the viral DNA load of lung and brain tissues between the ∆ORF37/38 strain-immunized groups contrasted with the ∆ORF38 strain-immunized group (*p* < 0.05) (Figure 3H). Histological observations revealed that lung and brain tissues from the ∆ORF37/38 10^8^ TCID_50_-immunized group showed no abnormalities (Figure 3H). However, in the ∆ORF38 10^7^ TCID_50_-immunized group, some hamsters exhibited exudate in the interstitium of the lungs and small amounts of neuronal necrosis and microglial activation in the brain tissues (Figure 3I,J). These results suggest that the ∆ORF37/38 strain has good potential as a vaccine and is effective in inducing host immunity against EHV-1 infection.

## 4. Discussion

The management of EHV-1 relies on a multifaceted approach involving vaccination, infection control measures, and proper management practices [35,36]. Despite the widespread administration of commercially available inactivated and modified live vaccines (MLVs) to horses by practicing equine veterinarians, outbreaks of EHV-1 disease persist [37,38]. Herpesviruses have developed various mechanisms facilitating host infection, including immune evasion, latent infection, and the induction of programmed cell death, which enhance virus survival and adaptation to the host environment [39,40]. Consequently, killed vaccines offer limited immune protection, while live attenuated vaccines that stimulate both humoral and cellular immune responses are preferred for effective defense against EHV-1 infection [41,42]. Live attenuated vaccine candidates have been developed utilizing ORF38-deletion mutants of EHV-1. However, reports have indicated that ∆ORF38 induces signs such as fever, headache, and asthenia in foals, induces pathological changes, and reduces body weight in test foals, which raises concerns regarding its safety [19,43,44]. To ensure the safety of the vaccine strain, it would be prudent to include another gene mutation that further attenuates virulence.

However, finding the ideal compromise between the degree of attenuation and immunostimulatory potential is a substantial challenge [45]. While viruses lacking multiple virulence factors may be viewed as a safer alternative, they run the risk of being excessively attenuated and failing to sufficiently stimulate the immune system [46]. Therefore, the precise deletion of virulence factors is crucial. The EHV-1 ORF37 gene encodes a protein present in the UL24 family of herpesviruses [47]. Although the function of this protein has been extensively researched in other alphaherpesviruses, such as HSV-1 and PRV, investigations of the EHV-1 ORF37 gene have been limited [47,48]. A study conducted by Kasem S et al. demonstrated that the deletion of ORF37 in the neuropathogenic strain Ab4 (ORF30 D752) impacted neuropathogenicity in a CBA mouse model of encephalitis [17]. Our previous study revealed notable differences in the amino acid sequences encoded by the ORF37 gene between this strain and YM2019 (ORF30 N752). However, the ∆ORF37/38 strain exhibited a lack of ability to induce significant respiratory and neurological signs and pathological damage in hamsters when administered at high doses, in contrast to the YM2019 strain, which resulted in complete mortality at equivalent doses. Notably, the ∆TK strain caused typical pathological damage in the brain tissues of hamsters at the same dosage, suggesting a substantial reduction in the virulence of the double-mutant strain, particularly regarding neuropathogenicity.

Moreover, the initial interactions between the host and pathogen at the site of virus entry, as well as mucosal immunity, play a significant role in the defense against EHV-1 [49]. Therefore, strong mucosal immunity is essential for preventing EHV-1 infection and decreasing the occurrence of EHM outbreaks. Intranasal vaccination is an effective approach for targeting the rapidly evolving mucosal immune system in the upper respiratory tract [50]. Transnasal administration of live attenuated vaccines often leads to the induction of efficient antibody and T-cell responses in immunized animals [51]. In this study, intranasal inoculation with ∆ORF37/38 elicited robust humoral and cellular responses. In a lethal challenge test with the YM2019 strain, an immune dose of only 10^6^ TCID_50_ per mouse resulted in 100% protection, with no lesions observed in the 10^8^ TCID_50_ immune dose group. The higher dose group exhibited greater protective efficacy. This evidence suggests that YM2019 ∆ORF37/38 has the potential to be developed as a live attenuated vaccine candidate. Despite the effects of the deletion of the ORF37/38 genes on viral replication during in vitro reproduction, resulting in a modest decrease in maximum viral titers, the growth kinetics of the mutant remained comparable, and there was no significant increase in the production costs for the vaccine when the virus was propagated in vitro. Furthermore, the ∆ORF37/38-eGFP strain may also be employed as a herpesvirus vector in the development of a vaccine. The deletion of the ORF37/38 genes may represent a promising avenue for the construction of a live attenuated vaccine.

During the initial infection, the virus establishes a latent infection in peripheral lymphocytes of the nasal mucosa, ganglia, and respiratory-associated lymphoid tissues, allowing it to evade the immune system’s attacks. There are no signs until stress triggers its reactivation and causes various diseases [52,53]. This makes it difficult to identify and remove infected animals from healthy horse herds in time. This reactivation elevates the likelihood of EHM in horses and the potential for viral transmission to other individuals through extensive viral shedding [54]. The biomolecular mechanisms controlling latent EHV-1 infection, despite their importance for the prevention and control of EHV-1 infection, are only partially understood [55]. Dexamethasone has been utilized as a potent inducer of latent herpesvirus infection in various studies [30,56]. Our current investigation revealed that dexamethasone effectively triggered latent EHV-1 infection. Interestingly, in the presence of dexamethasone, the ΔORF37/38 strain is unable to achieve high viral titers in brain tissue or cause neurological signs and tissue damage in hamsters, whereas the ΔORF38 strain succeeds in reactivating. This finding underscores the need for further investigation in horses to clarify the exact mechanisms by which the ∆ORF37/38 mutant affects the latency and reactivation of EHV-1. Moreover, measuring the infectious viral titer shed is crucial for any study assessing latency and reactivation and should be a key metric in further research. Monitoring signs of latent infection in animals reinfected after vaccination also warrants further investigation.

In summary, our preliminary research suggests that the EHV-1 YM2019 ∆ORF37/38 strain does not elicit noticeable respiratory or neurological signs in Syrian hamsters. Furthermore, unlike the ∆ORF38 strain, it does not achieve high viral titers in the lung and brain tissues of hamsters following dexamethasone stimulation, thereby significantly enhancing the safety profile of this live attenuated vaccine candidate. Immunization with the ∆ORF37/38 strain induces a more robust humoral and cellular immune response against EHV-1 compared to the ORF38 strain, resulting in complete protection (100%) against lethal challenge with the parental strain. These findings suggest the potential utility of YM2019∆ORF37/38 as a promising live attenuated vaccine candidate for combating EHV-1 infection.

## Data Availability

The original data presented in the study are openly available in National Center for Bio- technology Information (NCBI) GenBank at [accession number: MT063054].

## Supplementary Materials

The following supporting information can be downloaded at: https://www.mdpi.com/article/10.3390/v16091472/s1, Table S1: Primers used in this study; Table S2: Virus strains and plasmids used in this study; Table S3: Histopathologic grading of lung and brain tissues in EHV-1; Table S4: Scoring system for clinical signs in EHV-1; Figure S1: Construction and identification of an ORF38 gene-deletion mutant virus (EHV-1 ΔORF38). Figure S2: Construction and identification of ORF37/38-gene-deletion mutant virus (EHV-1 ΔORF37/38). Figure S3: Clinical signs of Syrian hamsters infected with EHV-1. Figure S4: Gross pathology of lung and brain of Syrian hamsters infected with EHV- 1 YM2019 strain.
