# Supplementary material for: Breaking Latent Infection: How ORF37/38-Deletion Mutants Offer New Hope against EHV-1 Neuropathogenicity
Source: Viruses. 2024 Sep 16;16(9):1472. doi: 10.3390/v16091472 (PMC11437417; doi:10.3390/v16091472)
Supplement: Supplementary file 1 [file viruses-16-01472-s001.zip › viruses-3138240-Supplementary.pdf]

# Supplementary Material

## 1 Supplementary Tables

**Supplementary Table S1. Primers used in this study.**

| Primer name | Sequence (5'-3') | Purpose |
|---|---|---|
| ORF38 L-F | GTGTTGGAGCTGACGG | Upstream homologous arm of ORF38 gene |
| ORF38 L-R | GCGAGCCGCCATTGA | |
| ORF38 R-F | ATCCCTCATACCGCCTC | Downstream homologous arm of ORF38 gene |
| ORF38 R-R | CCTGGAGATCCTGCTCA | |
| ORF37 R-F | GTGTTGGAGCTGACGG | Downstream homologous arm of ORF37/38 gene |
| ORF37 R-R | CTAAATCGACAAGGAG | |
| CMV-eGFP L-F | TAGTTATTAATAGTAATCAA | CMV-eGFP expression cassette |
| CMV-eGFP R-R | ATGCAGTGAAAAAAATGCT | |
| ΔORF38-F | TCTATACTTTCCTGAGC | Verification of ORF38 gene deletion |
| ΔORF38-R | AGACTCCACGGTTGC | |
| ΔeGFP-F | ATGGTGAGCAAGGGCGAG | Verification of eGFP gene deletion |
| ΔeGFP-R | ATCTAGATCCGGTGGATC | |
| ΔORF37-F | CTAGTACGATCTCACCGC | Verification of ORF37 gene deletion |
| ΔORF37-R | CACAACCGCTTCTATAAC | |
| eGFP-sgRNA-F | GCGAGGGCGATGCCACCTA | sgRNA targeting eGFP gene |
| eGFP-sgRNA-R | TAGGTGGCATCGCCCTCGC | |
| ORF37-sgRNA1-F | TATTCCGCCAAACGTGATA | sgRNA targeting ORF37 gene |
| ORF37-sgRNA1-R | TATCACGTTTGGCGGAATA | |
| ORF38-sgRNA2-F | CTCGGCGTTGAAGGTGCGCG | sgRNA targeting ORF38 gene |
| ORF38-sgRNA2-R | CGCGCACCTTCAACGCCGAG | |

**Supplementary Table S2. Virus strains and plasmids used in this study.**

| **Strain Number** | **Genotype/Phenotype** | **Source** |
|---|---|---|
| YM2019 | Wild type EHV-1 | China General Microbiological Culture Collection Center<br>GenBank: MT063054 |
| YM2019 ΔORF38 | EHV-1 ORF38 gene-deleted virus | This study |
| YM2019 ΔORF37/38 | EHV-1 ORF37/38 gene-deleted virus | This study |
| HDR-ORF38-E | pUC-19-ORF38 HDR-eGFP | This study |
| HDR-ORF38 | pUC-19-ORF38 HDR | This study |
| HDR-ORF37/38-E | pUC-19-ORF37/38 HDR-eGFP | This study |
| HDR-ORF37/38 | pUC-19-ORF37/38 HDR-eGFP | This study |
| ORF38-sgRNA1 | pX330-ORF38-sgRNA1 | This study |
| ORF37/38-sgRNA1 | pX330-ORF37/38-sgRNA1 | This study |
| CMV-eGFP-sgRNA | pX330-CMV-eGFP-sgRNA | This study |

**Supplementary Table S3. Histopathologic grading of lung and brain tissues in EHV-1**

| **Histopathologic changes** | **Histopathologic grading** | | | |
|---|---|---|---|---|
| **Lung** | **Normal** | **mild infection** | **moderate infection** | **fatal infection** |
| Interstitial pneumonia | 0 | 1 | 2 | 3 |
| Fibrosis | 0 | 1 | 2 | 3 |
| Inflammatory exudates | 0 | 1 | 1 | 2 |
| Neutrophilic inflammation | 0 | 1 | 2 | 2 |
| Hemorrhage | 0 | 1 | 2 | 3 |
| **Brain** | **Normal** | **mild infection** | **moderate infection** | **fatal infection** |
| Nonsuppurative encephalitis | 0 | 1 | 2 | 3 |
| Neuronal necrosis | 0 | 1 | 2 | 3 |
| Macrophage infiltration | 0 | 1 | 2 | 2 |
| Microglial activation | 0 | 1 | 1 | 2 |
| Gliosis | 0 | 1 | 2 | 3 |

a score of < 3 indicates no typical pathological changes; 3 ≤ score < 5 indicates mild pathological changes; 5 ≤ score < 8 indicates moderate pathological changes; 8 ≤ score < 10 indicates severe pathological damage.

**Supplementary Table S4. Scoring system for clinical signs in EHV-1**

| Sign | | Score |
|---|---|---|
| **Mental State** | Active and alert | 0 |
| | Depressed and standing still | 1 |
| | Lying still, moves after stimulation | 2 |
| | Lying still, no response after stimulation | 3 |
| **Feeding and Weight** | Immediate foraging and hoarding, normal weight gain | 0 |
| | Reduced feeding, slowed or no weight gain | 1 |
| | Does not actively forage but eats when fed separately, weight loss | 2 |
| | Completely stops eating, rapid weight loss | 3 |
| **Discharges** | No nasal discharge | 0 |
| | Small amount of watery nasal discharge | 1 |
| | Large amount of mucopurulent nasal discharge | 2 |
| | Excessive discharge from mouth, nose, and eyes | 3 |
| **Neurological Signs** | Has normal resistance reflexes | 0 |
| | Standing still with involuntary head and forelimb tremors | 1 |
| | Supine convulsions | 2 |
| | Complete paralysis | 3 |
| **Respiratory Rate** | Normal respiration (< 80/min) | 0 |
| | Slightly increased rate (81~120/min) | 1 |
| | Severely increased rate (> 121/min) | 2 |
| | Respiratory distress, exhibiting oral breathing | 3 |
| **Death/ Total** | | **15** |

a score of < 3 indicates no clinical signs; 3 ≤ score < 5 indicates mild clinical signs; 5 ≤ score < 8 indicates moderate clinical signs; 8 ≤ score < 10 indicates severe clinical signs; a score of > 10 indicates fatal signs.

## 2 Supplementary Figures

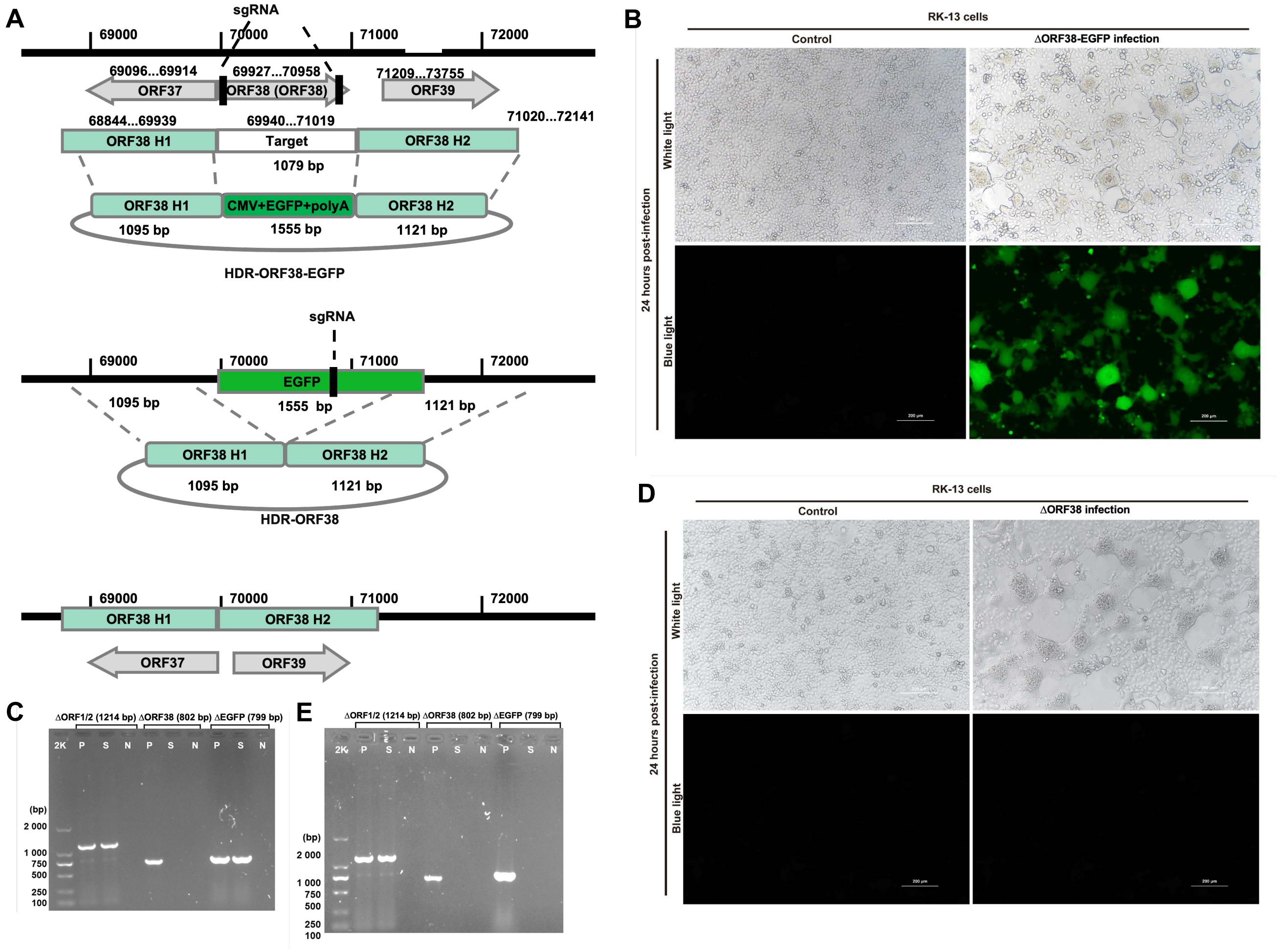


**Supplementary Figure S1.** Construction and identification of a ORF38 gene deletion mutant virus (EHV-1 ΔORF38). (A) The eGFP cassette was inserted using the Cas9 system based on the parental strain, and the ORF38 gene was replaced with the eGFP cassette to create the ΔORF38-eGFP mutant. (B) The CPE and green fluorescence were observed under a fluorescence microscope. (C) The deletion of the ORF38 gene and insertion of the eGFP gene were confirmed through PCR. (D) Under a fluorescence microscope, a CPE and no green fluorescence was observed. (E) PCR confirmed the deletion of the ORF38 and eGFP genes.

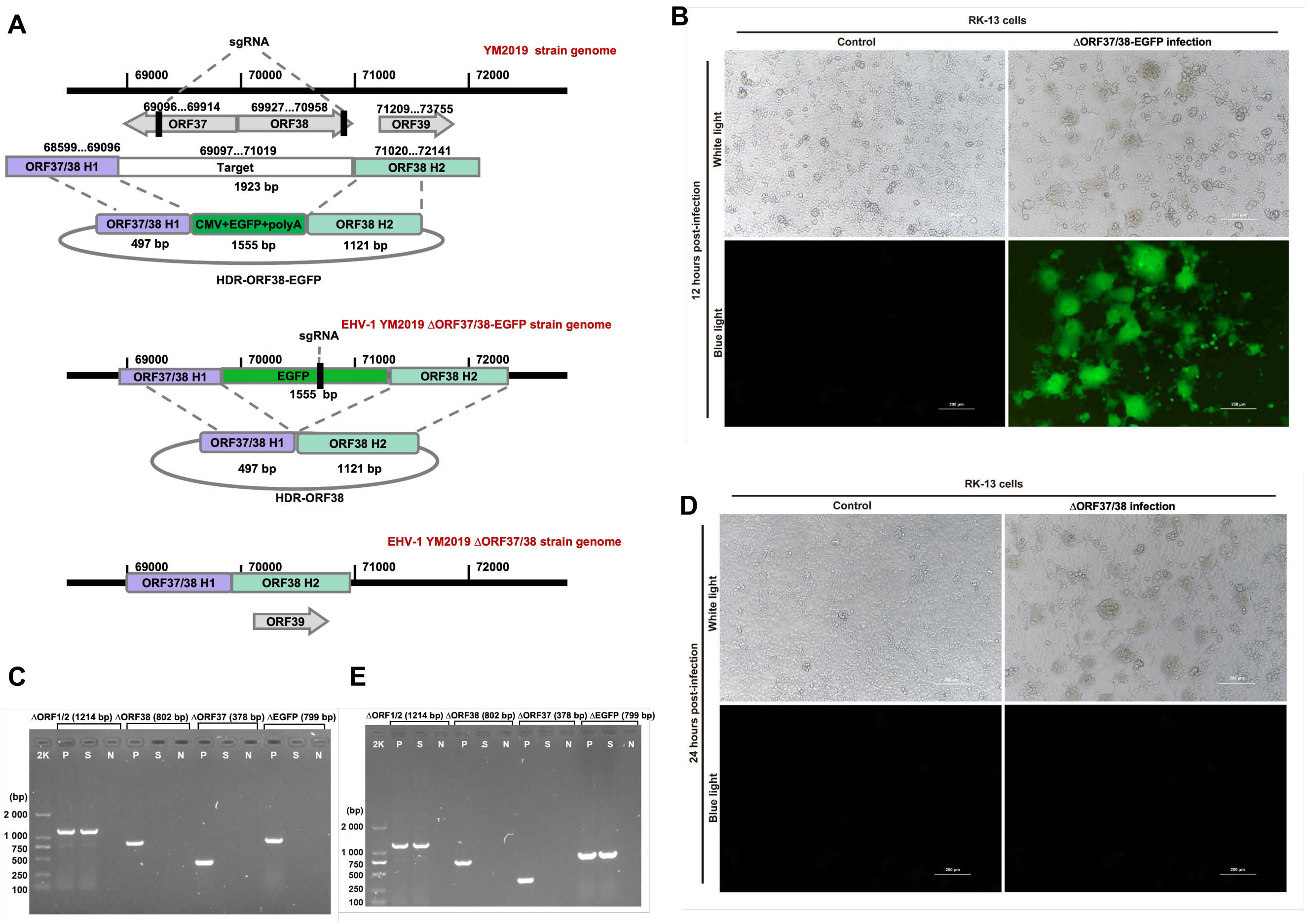


**Supplementary Figure S2.** Construction and identification of ORF37/38 genes deletion mutant virus (EHV-1 ΔORF37/38). (A) The eGFP cassette was inserted using the Cas9 system based on the Δgp2 mutant virus, and the ORF37/38 genes was replaced with the eGFP cassette to create the ΔORF37/38-eGFP mutant. (B) The CPE and green fluorescence were observed under a fluorescence microscope. (C) The deletion of the ΔORF37and ORF38 genes and insertion of the eGFP gene were confirmed through PCR. (D) Under a fluorescence microscope, a CPE and no green fluorescence was observed. (E) PCR confirmed the deletion of the ΔORF37, ORF38, and eGFP genes.

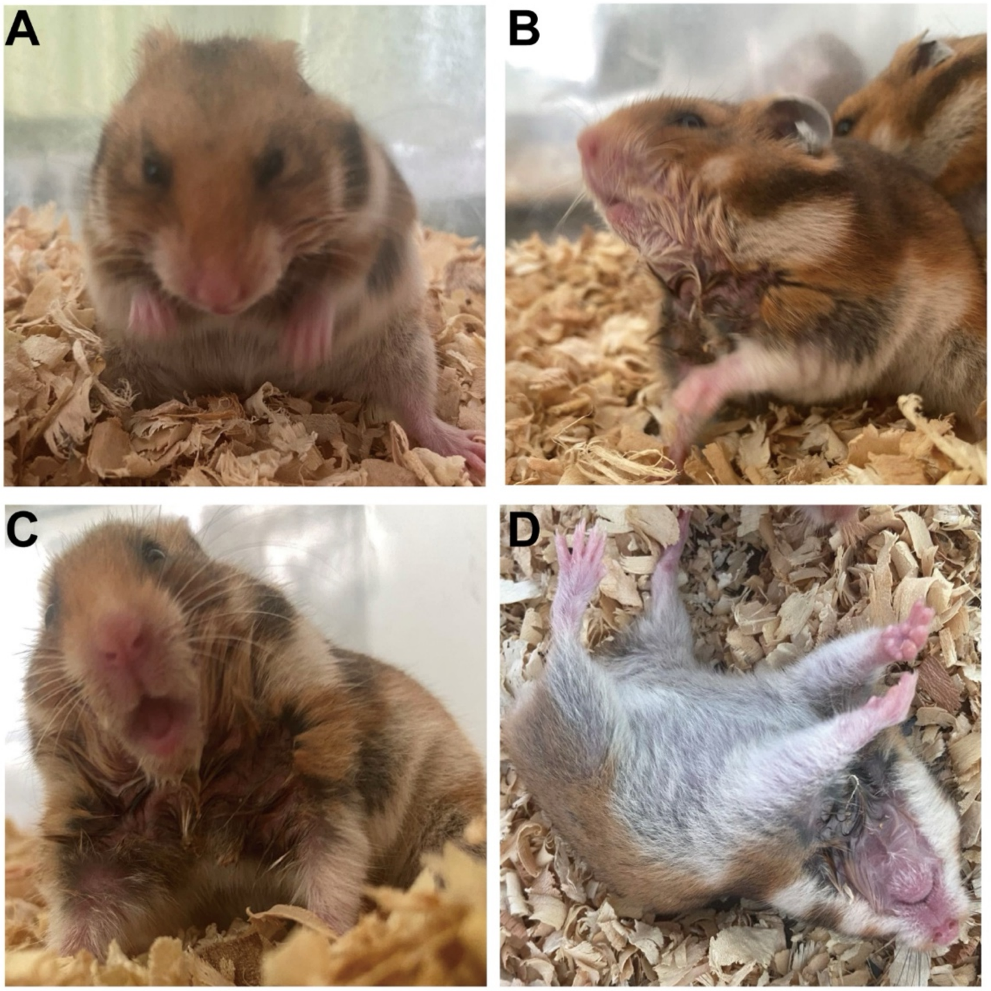


**Supplementary Figure S3.** Clinical signs of Syrian hamsters infected with EHV-1 YM2019 strain. (**A**) Mental depression and no appetite for food. (**B**) Salivation, (**C**) Breathing difficulties and choking, (**D**) Paralysis. For humanitarian reasons, all animals were euthanised at the end of clinical follow-up.

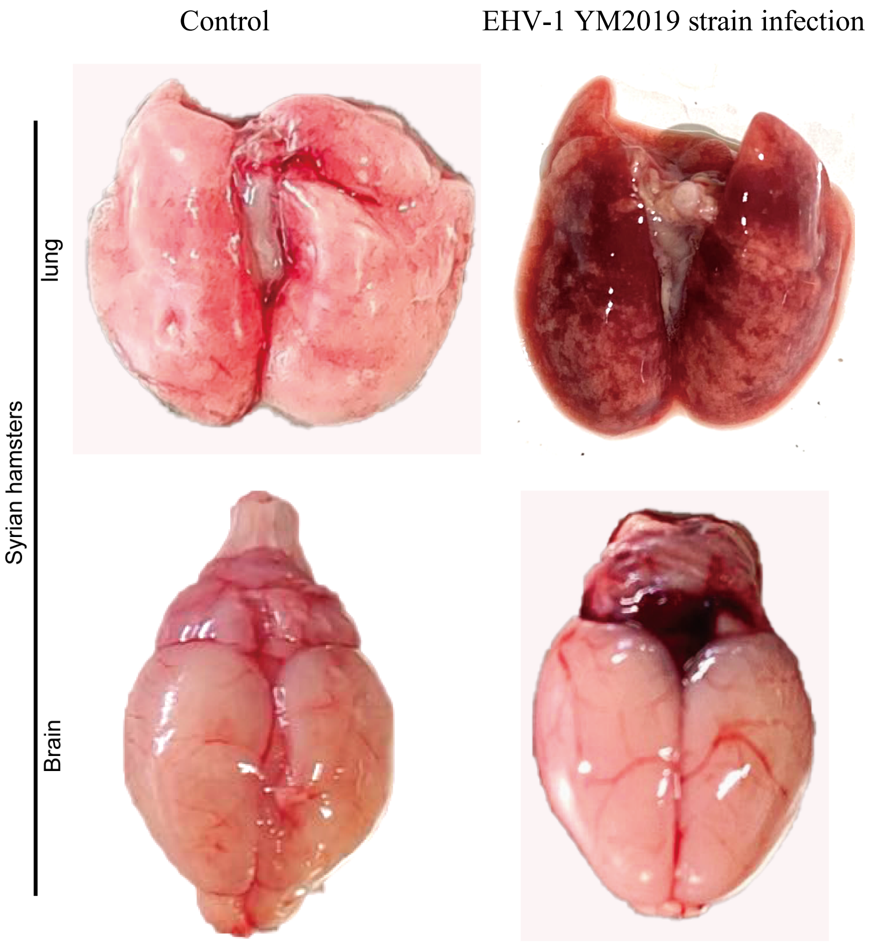


**Supplementary Figure S4.** Gross pathology of lung and brain of Syrian hamsters infected with EHV-1 YM2019 strain. Gross pathological observations of the lungs in hamsters infected with the EHV-1 YM2019 strain included pulmonary congestion and edema, with a mottled appearance on the surface, accompanied by exudation of fibrin and red blood cells. Gross pathological lesions in the brains of hamsters infected with the EHV-1 YM2019 strain presented as mild congestion and edema.
